# *Pepino mosaic virus* Infection of Tomato Affects Allergen Expression, but Not the Allergenic Potential of Fruits

**DOI:** 10.1371/journal.pone.0065116

**Published:** 2013-06-07

**Authors:** Saskia Welter, Sabine Dölle, Karola Lehmann, Dietmar Schwarz, Wolfram Weckwerth, Margitta Worm, Philipp Franken

**Affiliations:** 1 Department of Plant Nutrition, Leibniz-Institute of Vegetable and Ornamental Crops Großbeeren/Erfurt e.V., Großbeeren, Germany; 2 Department of Dermatology and Allergology, Charité-Universitätsmedizin, Berlin, Germany; 3 Proteome Factory AG, Berlin, Germany; 4 Department of Molecular Systems Biology, University of Vienna, Vienna, Austria; Institut Pasteur, France

## Abstract

The plant pathogen *Pepino mosaic virus* (PepMV) is a major disease of greenhouse tomato crops worldwide. Plant pathogens can induce expression of defence- or pathogenesis-related proteins, including identified allergens. Therefore we hypothesised that PepMV infection results in the expression of allergens leading to a higher allergenic potential of tomato fruits. Transcript level analyses showed differential expression of 17 known and putative tomato fruit allergen encoding genes at early and late time points after PepMV inoculation, but no general induction was detected. Immunoblot analyses were conducted and IgEs from a serum pool of tomato allergic subjects reacted with 20 proteins, of which ten have not yet been described. In parallel, skin prick tests with a group of tomato allergic subjects did not show a general difference between PepMV infected and non-infected tomato fruits and basophil activation tests confirmed these results. In summary, PepMV infection of tomato plants can lead to long-lasting up-regulation of particular allergens in fruits, but the hypothesis that this results in a higher allergenic potential of the fruits proved invalid.

## Introduction

The plant pathogen *Pepino mosaic virus* (PepMV), a highly infectious potex virus, is one of the major global diseases of greenhouse tomato crops [1]–[3]. Different PepMV isolates can cause a wide range of symptoms. Tomato fruits can be severely affected, leading to non-marketable fruits and associated high financial losses [4]–[8]. A strategy called cross-protection to protect plants against viral diseases was firstly described by McKinney in 1929 [9]. This strategy prevented disease symptoms caused by an aggressive strain of tobacco mosaic virus by pre-infecting the plant with a milder isolate causing no symptoms. Today cross-protection strategies are applied in commercial cultivation [10], including control of PepMV. Particularly PepMV infection at late stages of cultivation is assumed to be associated with a higher risk of severe symptoms [6], [8]. Therefore growers intentionally infect their tomato plants with mild strains of PepMV in order to minimize the effects of late natural infestation [11].

Despite the high economic impact of PepMV for tomato producers, the response of the plant was not analysed until recently, when a microarray analysis was conducted [12]. Approximately 4,000 tomato genes, including those encoding defence- or pathogenesis-related (PR) proteins, showed differential expression four days after infection with PepMV. Thereafter the number of regulated genes continuously declined up to 12 days after infection. This correlates with the observation that viral symptoms are often transient and plants can recover from the first infection shock. At later stages symptoms can reappear [13] and this can be accompanied by fluctuating defence responses, including differential expression of PR-proteins.

The activation of PR-proteins after a virus attack was identified a long time ago [14]. Typical representatives are 1,3-β-glucanases, chitinases, osmotin-like proteins and peroxidases [15], [16]. Interestingly, PR-proteins show high homologies to allergens and allergenic activity could be confirmed in many PR-protein families [17], [18]. For example, in apple an infection of young leaves from seedlings by fireblight, a bacterial disease, provoked an increase in the PR-protein and allergen Mal d 1.01 encoding gene [19].

The expression of defence-related or PR-proteins presenting allergens has also been shown in fruits of tomato [20]–[24]. Previously, we observed that gene expression of such proteins was induced in tomato fruits of plants harbouring symbiotic arbuscular mycorrhizal fungi in their roots; but this did not result in higher reactions of tomato allergic subjects in clinical allergy tests [25]. However, the impact of infectious pathogens on the allergenic potential of tomato fruits has not yet been analysed.

Tomato fruits commercially available in supermarkets are known to be naturally or intentionally infected with PepMV [26]. Considering this information, there is an urgent need to analyse these tomato fruits for allergen expression, and to investigate their allergenic potential during the harvest period of a PepMV infected tomato plant.

Three hypotheses were tested for the current study. First, the expression of genes encoding defence-related or PR-proteins with homologies to allergens are also expressed at much later time points after PepMV infection than previously reported [12]. Second, these genes are not only differentially expressed in leaves, but also in fruits infested by the virus. Third, tomato fruits infested with PepMV possess higher allergenicity than those that are virus-free.

To test the first two hypotheses, the expression of confirmed and putative allergens was analysed at two different time points after PepMV infection in fruits, and at one time point in leaves. In addition, new putative allergens were identified by immunoblot analyses and subsequent mass spectrometry. For the third hypothesis, standardised clinical allergy tests (skin prick tests and basophil activation/degranulation tests) were conducted on tomato allergic subjects. While our results agreed with the first two hypotheses, we saw no evidence for higher allergenicity of PepMV infected tomato fruits.

## Materials and Methods

### Plant Material

Tomato plants (*Solanum lycopersicum*), cultivar ‘Matina’ (Hild GmbH, Marbach a. N., Germany) were grown in a greenhouse from May until November 2010 at the Leibniz- Institute of Vegetable and Ornamental Crops in Großbeeren, Germany. Mean temperature, daily radiation, and humidity were 21.1°C, 28.9 mol m^−2^d^−1^ and 71.6%. Seeds had been disinfected with 4% MennoFlorades (Menno Chemie Vertrieb GmbH, Norderstedt, Germany) for 30 min to exclude any pre-infection with PepMV. At the seven-leaf stage plants were transferred to hydroponic cultivation conditions (nutrient solution in mM: 12 NO_3_, 4 K, 5 Ca, 0.1 NH_4_, 0.5 P, 2.2 Mg and 3.4 SO_4_, in µM: 50 B, 25 Fe, 5 Mn, 7 Zn, 0.7 Cu and 0.5 Mo).

Ten weeks after sowing half of the plants were inoculated with *Pepino mosaic virus* CH2 isolate (strain PCH 06/104, NCBI accession number DQ000985) by rubbing with an extract of PepMV infested tomato leaves on the second fully developed leaf. This extract was prepared by grinding infested leaf material in distilled water. Two weeks post inoculation (WPI) all plants were tested for a systemic PepMV infestation using an ELISA Kit (Agdia, Elkhart, Indiana, USA) according to manufacturers’ instructions. All non-infected plants were checked weekly following this procedure and always gave a negative response. Red-ripe tomato fruits (stage 10–11 of the colour screening scale for tomato, International standardisation of fruits and vegetables) were harvested at 3 and 10 WPI in liquid nitrogen, freeze dried and ground for RNA accumulation analyses. At 10 WPI the fifth youngest leaf of each plant was also collected and treated similarly. Each sample comprised four fruits or leaves and for each time point and treatment three samples were taken as replicates. For all other analyses a mush of ten tomatoes was directly used (skin prick test) or stored at −80°C until use (basophil activation test and protein gels). Skin prick tests were carried out with fresh material between the 4^th^ and 13^th^ WPI. Tomatoes for basophil activation tests and protein gels were harvested over the whole experimental period at 4, 8, 10, and 12 WPI.

### RNA Accumulation Analysis

Total RNA was extracted from freeze dried fruit or leaf material using TRIzol reagent (Invitrogen GmbH, Darmstadt, Germany) and treated with the RNase free DNase Set (Qiagen, Hilden, Germany). Genomic DNA contamination was excluded by RNA analysis with PCR. One µg RNA was reverse transcribed with an M-MLV reverse transcriptase system using oligo-dT primers (Promega, Mannheim, Germany). The resulting cDNA was used as a template in a 1∶100 dilution of a master mix containing 50% Power SYBR Green (Applied Biosystems, Warrington, UK) and 200 nmol/L of each primer (Table S1). Quantitative real time RT-PCR (qRT-PCR) was carried out using the 7,500 Fast Real-Time PCR System (Applied Biosystems, Warrington, UK) with the following temperature programme: 50°C for 2 min, 95°C for 10 min, 40 cycles of 15 s at 95°C, 1 min at 60°C followed by a melting curve analysis. qRT-PCR reactions were conducted in triplicate. Relative RNA accumulation rates were calculated using Biogazelle qBase Plus software [27] with three evaluated reference genes (geNorm, integrated in qBase Plus) for fruits (encoding *18S rRNA* (ribosomal RNA), *GAPDH* (glyceraldehyde 3-phosphate dehydrogenase), and *UBI* (ubiquitin)) and two for leaves (encoding *18S rRNA* and *GAPDH*) resulting in CNRQ (calibrated and normalised relative quantities) values. To analyse the expression of confirmed and putative tomato allergens, including defence-related proteins, 17 genes were selected for RNA accumulation analyses, according to previous immunoblots and the ‘Allergome’ database (www.allergome.org).

### Preparation of Tomato Protein Extracts

For immunoblotting tomato mush of ten tomatoes was mixed with extraction buffer (25 mM Tris/HCl pH 7.4, 50 mM KCl, 1.5 mM EDTA, 2.9 mM benzamidine, 2.1 µM leupeptine, 1 mM phenylmethylsulfonyl fluoride, 1 µM Pepsatin A) containing 7 M guanidinehydrochloride, centrifuged, and protein was precipitated from supernatant with ethanol over night at −20°C. The pellet was resuspended in extraction buffer containing 7 M urea, 2 M thiourea, 2% carrier ampholytes (pH 2–4; Serva, Heidelberg, Germany) and 70 mM DTT.

For basophil activation tests tomato mush was mixed with PBS buffer (137 mM NaCl, 10 mM Na_2_HPO_4_, 2.7 mM KCl, 2 mM KH_2_PO_4_, pH 7.4) containing 5% NP40, centrifuged, and protein was precipitated from supernatant with acetone over night at −20°C. The pellet was resuspended in PBS with protease inhibitor mix (complete mini-EDTA free tablets, Roche, Indianapolis, USA).

Protein concentrations were determined by Bradford assays (Proteome Factory AG, Berlin, Germany) [28].

### 2D Gel Electrophoresis, Immunoblotting, Identification, and Quantification of Putative Allergens

Immunoblot analyses were carried out with protein extracts from ten pooled infected or non-infected fruits in three technical replicates. Two dimensional electrophoresis (2DE) was conducted following the protocol by Klose and Kobalz [29]. Isoelectric focussing (first dimension) was performed in vertical rod gels containing 9 M urea, 4% acrylamide, 0.3% piperazine diacrylamide, 5% glycerine, 2% carrier ampholyte (pH 2-11), 0.06% TEMED, 0.08% ammonium persulfate. 60 µg of protein extract was focussed at 1,841 V. SDS–PAGE (second dimension) was performed in gels (0.1 cm×7 cm×8 cm; 15% acrylamide, 0.2% bisacrylamide, 375 mM Tris-HCl (pH 8.8), 0.1% SDS, 0.03% TEMED, and 0.08% ammonium persulfate). 2DE separations were performed in duplicate. One gel was stained with FireSilver (Proteome Factory, Berlin, Germany) for preparative applications; the other gel was used for immunoblotting. 2DE gels were blotted using an Immobilon-P membrane (PVDF, pore size 0.45 mm; Millipore, Bedford, USA) and a Trans-Blot SD Semi-Dry Transfer Cell (Biorad, Munich, Germany) at a constant current and 5 V over night at 4°C.

After washing and blocking, membranes were incubated with a serum pool of nine tomato allergic subjects (diluted 1∶10 in TBS Tween containing 1% (w/v) BSA) over night and then incubated with peroxidase conjugated goat anti-human IgE (Sigma, Taufkirchen, Germany, diluted 1∶3,000 in TBS Tween containing 1% (w/v) BSA) for 2 h. Immunoblots were developed with Pierce ECL Western Blotting Substrate (Thermo Fisher Scientific, Rockford, USA). Between all incubation steps the membrane was washed with TBS Tween (5 times for 10 min). Proteins were identified by Proteome Factory AG (Berlin, Germany). After in-gel digestion with trypsin, peptides were analysed in a nanoLC-ESI-MS/MS. Proteins were identified using MS/MS ion search of the Mascot search engine (Matrix Science, London, England) and a protein database (National Center for Biotechnology Information (NCBI), Bethesda, USA). MASCOT expresses the probability that peptides match at random to a given protein by a probability score. A score larger than 57 indicates identity or extensive homology (p = 0.05).

Separate 2DE protein gels (20×30 cm) were used to quantify representative putative tomato allergens at the protein level. Identification and quantification was achieved by comparison with immunoblots. The 2DE gels were digitised at a resolution of 150 dpi using a PowerLook 2100XL scanner with transparency adapter. 2D image analysis and protein spot quantification was performed using the Proteomweaver software 3.1 (Definiens AG, Munich, Germany).

### Tomato Allergic Subjects/Ethics Information

All subjects were recruited at the Allergy-Center-Charité, Berlin, Germany, between September and November 2010. Only subjects with a positive history of adverse reactions to tomato were included. The study was approved by the local ethics committee (Ethikkommission Charité-Universitätsmedizin Berlin, EC-No. 1832/Si.258) and all subjects gave written informed consent prior to the investigations. Subjects’ characteristics are listed in Table S2.

### Skin Prick Tests

Freshly prepared tomato mush of ten red-ripe tomatoes, harvested on the same day as the test or the day before, was used. Skin prick tests were performed according to the recommendations of GA^2^LEN [30] performed as prick-to-prick. Histamine dichloride (10 mg/mL, ALK-Abelló, Wedel, Germany) and saline solution (pH 7.4, ALK-Abelló) served as positive and negative controls. The skin reactions were considered positive when the wheal diameter was ≥3 mm after 15 min in the absence of a reaction towards the negative control.

### Basophil Activation and Degranulation Tests

Blood of five tomato allergic subjects was investigated in a basophil activation and degranulation test. Tomato protein extracts in different concentrations (0.005, 0.05, 0.5, 5, 50, 500 µg/mL) as well as a positive (5 µg/mL human anti-IgE, Biozol HP6061, Eching, Germany) and a negative (medium/10% foetal calf serum) control served for stimulation of the cells at 37°C for 15 min. Staining was performed as previously described [31]. Activation and degranulation were determined by flow cytometry using MACS Quant Analyser (Miltenyi Biotec, Bergisch Gladbach, Germany) and data were analysed using FCS Express V3 software (De Novo Software, Los Angeles, CA, USA). CD3 negative and CCR3 positive cells were considered as basophils. Raw data were normalised relative to the positive control. Activation was determined by %CD203c^+^, whereas degranulation was determined by %CD63^+^ basophils.

### Statistics

Statistical analyses were carried out using Statistica (version 9, Tulsa, OK, USA). RNA accumulation analyses were subjected to one-way analysis of variance (ANOVA) procedures (p = 0.05). The skin prick test and basophil test data were subjected to the non-parametric test. Medians were separated by the Mann-Whitney U test procedure (p = 0.05).

## Results

### PepMV Infection

Tomato plants were successfully infected with PepMV as confirmed by ELISA and quantitative real time RT-PCR (qRT-PCR) two weeks post inoculation (WPI, data not shown). Virus titres of leaves and fruits were compared at 10 WPI and revealed higher PepMV accumulation in fruits than in leaves. Comparison of viral accumulation in fruits at the two harvest time points showed higher titres at 3 than at 10 WPI (Figure S1). Interestingly, plants showed typical PepMV symptoms on fruits (marbling) at 3 WPI in contrast to 10 WPI, when not a single sign was left on the fruits (Figure S2).

### Expression of Allergen Encoding Genes

**Figure 1:**
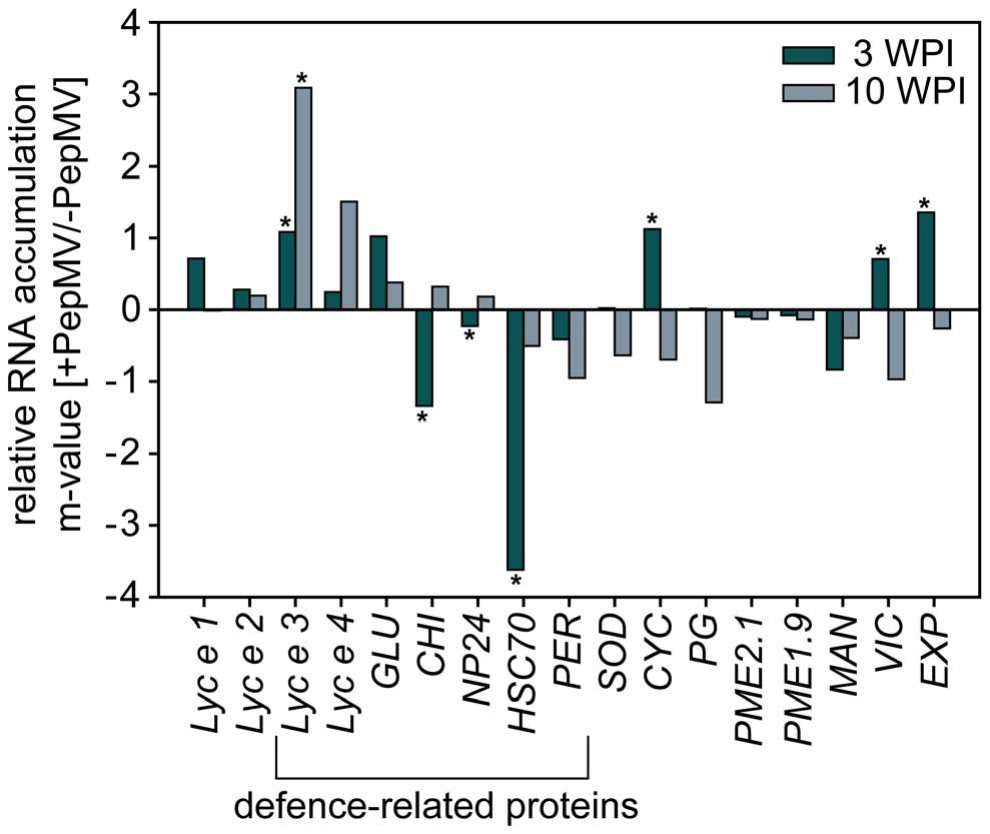
Relative RNA accumulation of known and putative allergen encoding genes in tomato fruits. RNA was extracted from tomato fruits of PepMV infected and non-infected plants 3 and 10 weeks after inoculation (WPI). qRT-PCR analyses were carried out with primer pairs for genes encoding the following proteins: Defence-related proteins: *Lyc e 3*: lipid-transfer-protein; *Lyc e 4*: from pathogenesis-related protein family PR-10; *GLU*: 1,3-β-glucanase; *CHI*: chitinase; *NP24*: thaumatin-like protein, osmotin precursor; *HSC70*: heat shock protein cognate; *PER*: peroxidase. Other confirmed and putative allergens: *Lyc e 1*: profilin; *Lyc e 2*: β-fructofuranosidase; *SOD*: superoxide dismutase; *CYC*: cyclophilin; *PG*: polygalacturonase; *PME2.1*: pectinmethylesterase 2.1; *PME1.9*: pectinmethylesterase 1.9; *MAN*: mannosidase; *VIC*: vicilin; *EXP*: expansin. Data were analysed with qBase software and calculated with CNRQ values. Target genes were normalised with the geometric mean of three reference genes (*18S rRNA*, *GAPDH,* and *UBI*). Data are given in m-values (log_2_ (CNRQ +PepMV/CNRQ -PepMV)). Significant differences between PepMV infected plants and non-infected controls are indicated by asterisks (one-way ANOVA, p = 0.05; n = 3).

To analyse the expression of confirmed and putative tomato allergens, including defence-related proteins, 17 genes were investigated for RNA accumulation using qRT-PCR. While four genes were significantly up-regulated and three down-regulated in PepMV infected tomato fruits at 3 WPI, only one gene was induced by the virus at 10 WPI (Figure 1). At 10 WPI leaves were also analysed for RNA accumulation of certain defence-related and allergen encoding genes. At this late time point, five among ten genes showed more than two-fold virus-enhanced RNA accumulation (Figure S4).

### Putative Allergens Identified via Immunoblot Analyses and Representative Proteins Quantified

**Figure 2:**
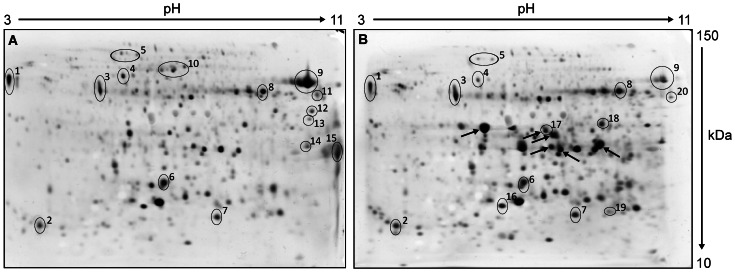
2DE protein gels identify putative tomato allergens. Proteins were extracted from non-infected (A) and PepMV infected (B) tomato fruits at 10 WPI. Proteins detected in immunoblots with the sera from nine tomato allergic subjects and identified by mass spectrometry are marked with numbers and listed in Table 1. Arrows highlight PepMV coat protein identifications.

To detect new putative allergens that might arise in tomato fruits infected with PepMV, immunoblot analyses with a serum pool of nine tomato allergic subjects were conducted as three technical replicates. Among those proteins that reacted with the serum pool, 20 could be identified by mass spectrometry. Nine of the putative allergens occurred in protein extracts from both infected and non-infected fruits, while five or six were only present in extracts from infected or non-infected fruits, respectively (Table 1). Among these, not only confirmed allergens such as Lyc e 1, Lyc e 2, and known putative allergens such as polygalacturonase, peroxidase, and glucanase (www.allergome.org), but also newly identified proteins were found, for example heat shock proteins and mannosidase (Figure 2).

**Table 1:** Putative allergens identified in PepMV infected and non-infected tomato fruits.

spot no. in gel	name	NCBI accession number	molecular weight/isoelectric point	score/sequencecoverage [%]	known as allergen in tomato
**putative allergens identified in PepMV infected (+PepMV) and non-infected (−PepMV) fruits**					
2	profilin	gi|17224229	14.1/4.7	−127/24 (+137/43)	yes, Lyc e 1
4	acid β-fructofuranosidase	gi|124701	70.1/5.5	−1221/28 (+424/27)	yes, Lyc e 2
9	polygalacturonase-2	gi|129939	50/6.4	−1707/52 (+669/41)	yes, PG
1	suberization-associated anionic peroxidase 1	gi|129807	38.7/4.9	−886/26 (+357/19)	yes, PER
3	1-aminocyclopropane-1-carboxylate oxidase homolog	gi|119640	41.1/5.6	−219/25 (+131/17)	no
8	fructose-1,6-bisphosphate aldolase	gi|14484932	36.5/8.7	−1333/55 (+283/74)	no
7	nucleoside diphosphate kinase	gi|575953	15.4/6.8	−422/40 (+347/40)	no
5	heat shock 70 kDa protein, mitochondrial	gi|585273	73/6.4	−606/24 (+n.d.)	no, HSC70
6	small heat shock protein	gi|4836469	17.7/5.8	−436/54 (+555/65)	no
**putative allergens only identified in non-infected fruits (−PepMV)**					
12	glucan endo-1,3-β-glucosidase B	gi|461979	39.7/7.9	333/37	yes, GLU
13	vicilin	gi|166053040	66.1/8.2	241/12	yes, VIC
14	pathogenesis-related protein PR P23	gi|19315	25.1/6.1	102/8	yes
15	NP24 protein precursor	gi|170467	25.7/8.3	180/27	yes, NP24
11	mannan endo-1,4-β-mannosidase 4	gi|125951563	45.3/8.9	331/24	no, MAN
10	enolase	gi|119354	47.8/5.7	1463/51	no
**putative allergens only identified in PepMV infected fruits (+PepMV)**					
17	ascorbate peroxidase	gi|21039134	42.1/8.7	234/21	yes
18	basic 30 kDa endochitinase	gi|544011	34.3/6.2	381/48	yes, CHI
20	polygalacturonase inhibitor protein	gi|469457	36.5/8.7	305/29	no
19	abscisic stress-ripening protein 1	gi|584786	13.1/6.8	159/22	no
16	superoxide dismutase [Cu-Zn], chloroplastic	gi|134682	22.2/5.8	293/34	no, SOD

Protein extracts from tomato fruits were separated by 2DE gel electrophoresis and analysed by immunoblotting with an IgE serum pool from nine tomato allergic subjects. Spots showing a reaction were eluted from parallel gels and identified using mass spectrometry. Mascot searches were done in 08/2011. Spots listed by numbers as identified in Figure 2. Abbreviations: −: −PepMV; (+): +PepMV; n.d.: not detectable with mass spectrometry; Lyc e 1 and Lyc e 2: confirmed tomato allergens by the International Union of Immunological Societies (IUIS).

**Figure 3:**
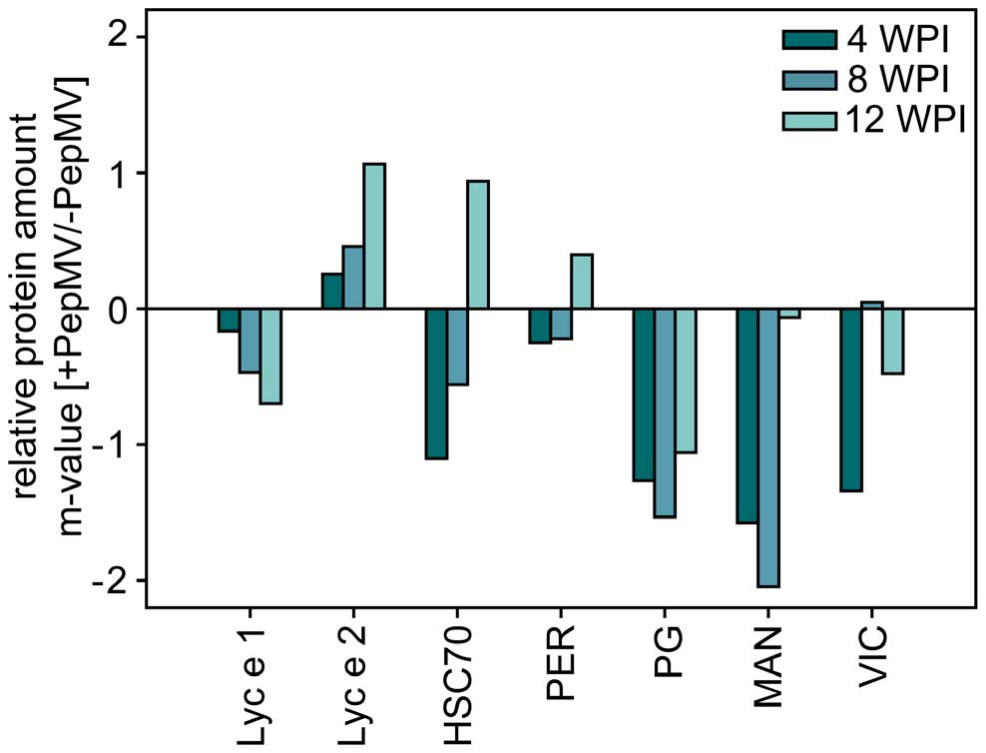
Quantification of known and putative tomato allergens at the protein level. Proteins were extracted from PepMV infected and non-infected control fruits at 4, 8 and 12 weeks post inoculation with PepMV (WPI) and separated on 2D gels. Seven proteins could be identified and quantified based on comparison with immunoblots. Proteins were quantified due to their spot intensity on the gel using Proteomweaver software. Lyc e 1: profilin; Lyc e 2: β-fructofuranosidase; HSC70: heat shock protein cognate; PER: peroxidase; PG: polygalacturonase; MAN: mannosidase; VIC: vicilin. Data are given in m-values (log_2_ (+PepMV/−PepMV)).

Seven representative proteins were quantified on separate 2DE protein gels of PepMV infected and non-infected control tomato fruits from 4, 8, and 12 WPI. The overall protein pattern looked very similar at different time points and only minor differences could be detected. Except Lyc e 2, most putative tomato allergens were slightly down-regulated in PepMV infected tomato fruits (Figure 3). Expression of the heat shock protein (HSC70) and peroxidase (PER) differed between the three investigated time points.

### Clinical Allergy Tests

**Figure 4:**
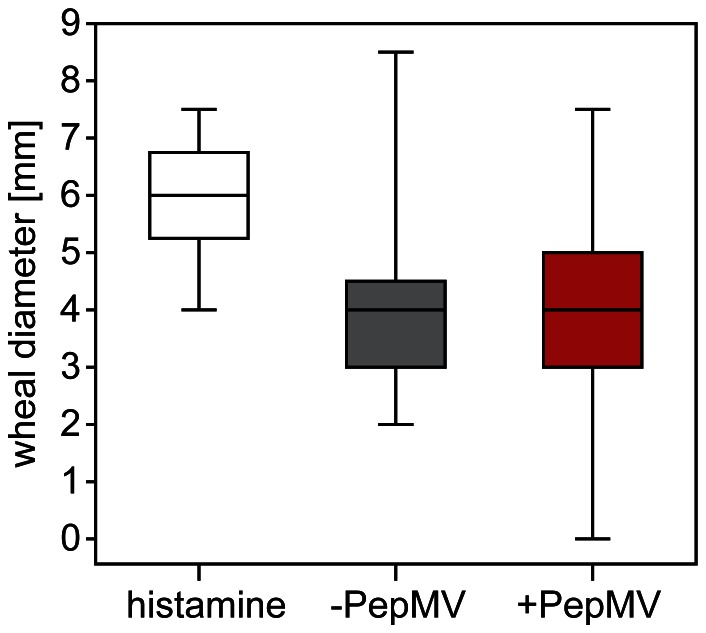
Skin prick tests of tomato allergic subjects with PepMV infected and non-infected control fruits. Tests were carried out on nine subjects using tomato fruit mush from PepMV infected and non-infected control plants during the 4^th^–13^th^ week post inoculation (WPI). Histamine dichloride (10 mg/mL) was used as a positive control. The median is depicted as a black line. No significant difference was found (Mann-Whitney U test, p = 0.05; n = 9).

**Figure 5:**
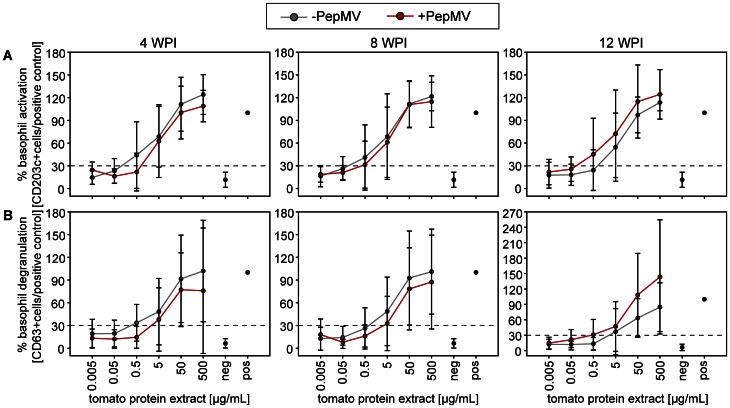
Basophil activation and degranulation tests of tomato allergic subjects with PepMV infected and non-infected fruits. Dose response curves of basophil activation (A) and degranulation (B) with tomato fruit protein extract from 4, 8 and 12 weeks post inoculation with PepMV (WPI). Basophil activation and degranulation is shown in %CD203c^+^ and %CD63^+^ cells normalised to positive controls. The mean of five tomato allergic subjects is shown. No statistical significant differences were found (Mann-Whitney U test, p = 0.05; n = 5).

Skin prick tests on nine tomato allergic subjects showed a positive reaction to the applied tomato mush, but the reaction intensity was highly variable (Figure S5). In summary, no significant difference could be detected between the reactions to PepMV infected and non-infected tomato fruits (Figure 4). Subsequently, basophil activation and degranulation tests were carried out with blood from five subjects using tomato protein extracts from three different time points (4, 8, and 12 WPI). Basophil activation and degranulation increased in a dose-dependent manner (Figure 5 and Figure S6). However, basophil activation or degranulation gained with 5 µg/mL protein extract revealed neither a significant difference between the reactions to PepMV infected and non-infected control extracts, nor between the extracts from fruits harvested at different time points (Figure S7). Individual variation was as high as in the skin prick tests. Anyway, at 4 and 8 WPI a lower amount of tomato protein extract of non-infected tomatoes was necessary to activate (AC_30_ [µg/mL]: 4 WPI: −PepMV: 0.2, +PepMV: 1.4; 8 WPI: −PepMV: 0.2, +PepMV: 0.4) or degranulate (DC_30_ [µg/mL]: 4 WPI: −PepMV: 0.4, +PepMV: 3.5; 8 WPI: −PepMV: 1.3, +PepMV: 4.3) 30% of the basophils. At 12 WPI this was inverted and a lower amount of protein extract of PepMV infected tomatoes was necessary (AC_30_ [µg/mL]: −PepMV: 1.3, +PepMV: 0.1; DC_30_ [µg/mL]: −PepMV: 3.7, +PepMV: 0.5).

## Discussion

Tomato fruits represent an important part of human diet and possess many health-related compounds [32], [33]. Unfortunately, a certain percentage of the population cannot consume this important vegetable, because they suffer from local and systemic allergic reactions [34]. This study aimed to answer the question of whether infection of tomato plants with *Pepino mosaic virus* (PepMV) influences their allergenic potential. One particular aspect of our experiments is that we analysed defence responses of tomato fruits, rarely investigated in plant pathogen interaction studies. Interestingly, we could detect that PepMV particles accumulated at higher abundance in these fruits, probably because they are major sink organs. Secondly, we analysed major greenhouse grown tomato plants weeks after inoculation with the virus. Cultivation of plants under conditions similar to commercial cultivation in horticulture, and analyses of tomato allergy relevant fruits, highlight the difference to former studies, which were performed mainly on vegetative organs. Additionally, this study provides new insights into plant defence response to viruses.

### Allergen Encoding Gene Expression after PepMV Infection Differs between Time Points and Plant Organs

One hypothesis of this study was that expression of genes encoding defence-related and PR-proteins that interact with the IgEs of tomato allergic subjects is influenced by the spread of PepMV in the plants. Contrary to our expectations and other reports, e.g. where resistance against *Botrytis cinerea* was increased after PR-proteins were induced in tomato fruits [35], no general up-regulation of PR-genes was detected in fruits of PepMV infected plants. Greater differences in RNA accumulation of the investigated genes were observed between time points 3 and 10 WPI (Figure S3). This might be explained by a fluctuation in the plants’ defence response over time, which would be consistent with the severe symptoms see on fruits at 3 WPI, but their absence at 10 WPI (Figure S2).

Interestingly, virus titres were also significantly higher at 3 WPI. Hanssen and Thomma [1] also reported a common recovery from PepMV symptoms after initial infection, and later suggested a relationship with the observed transient transcriptomic response [12]. Furthermore, plant pathogen interaction or the expression of PR-proteins in general might also be influenced by plant age and surrounding environmental conditions [36]–[40]. Former studies reported differences in PR-protein RNA accumulation after virus attack [3], [41]–[43], but mostly they refer to hours or a few days after the infection. Such early responses have also been observed in young tomato plants infected with PepMV [12]; but one has to consider that these studies focused on leaf material. In leaves we could also observe that most investigated defence-related genes showed higher RNA accumulation at 10 WPI, even if induction levels were weak.

It appears that after an initial strong response of the plant to PepMV infection [12], adaptation to the permanent presence of the virus leads to constitutive expression of numerous defence-related genes. This low-level expression could be part of systemic acquired resistance of plants to prevent a further attack by biotrophic pathogens [44]. These differences in PR-protein and allergen encoding gene expression between leaves and fruits as well as between young and major plants have already been shown for different apple cultivars infected with fireblight [19]. The authors showed an up-regulation of major apple allergen *Mal d 1.01* transcripts in leaves from seedlings after fireblight infection. In contrast, an increase in this PR-10 protein could not be confirmed in apple fruits of major trees. Together, these findings might be considered in further descriptions about the defence response of a plant and the impact of pathogen infection on the allergenic potential.

### Tomato Allergic Subjects’ Sera Reacted Differently to PepMV Infected Tomato Fruit Protein Extracts Compared to Non-infected Controls, Revealing New Putative Tomato Allergens

All putative tomato allergens identified by immunoblots of PepMV infected tomato fruits with sera from tomato allergic subjects’ belonged to proteins involved in stress or defence responses of the plant. Besides two already known tomato allergens, chitinase [20] and anionic peroxidase [23], three others (polygalacturonase inhibitor protein, abscisic stress-ripening protein and superoxide dismutase) were identified and described here as new putative tomato allergens. This might be explained by induced plant defence and a higher expression of these proteins in infected fruits.

Conversely, the subjects’ sera reacted with other PR-proteins (glucanase, NP24 and PR23) on the corresponding immunoblots of non-infected tomato fruits. This might be explained by constitutive expression of some PR-proteins, especially in fruits that are more likely to be attacked by insects or fungi [18]. Additionally, plants cultivated in the greenhouse under commercial growing conditions are not fully protected against other pathogens, e.g. white fly [45], [46] and non-optimal environmental conditions [39]. Therefore, PR-proteins may also be induced in non-infected control plants. Moreover, the alkaline proteins glucanase, vicilin, NP24 and PR23 are not even visible on protein gels of PepMV infected tomato fruits (Figure 2). Despite performing the immunoblots and corresponding gels in three replicates, technical problems, for example partial damage of the porous basic ends of the first dimension gels, cannot be completely excluded. Therefore, these alkaline proteins can be described as IgE reactive proteins, but should be excluded from comparison of reactions to PepMV infected and non-infected control fruit proteins.

Lyc e 1, Lyc e 2, and polygalacturonase, three of the major tomato allergens [47], evoked comparable reactions with the subjects’ serum pool on immunoblots with PepMV infected tomato fruits and control blots, even if they looked differentially expressed on 2D protein gels (Figure 2 and 3). Lyc e 3, another confirmed tomato allergen, could not be detected in the current immunoblot analyses. Lyc e 3 is a lipid-transfer-protein well known in plant defence responses [41] and RNA accumulation was significantly enhanced at both time points of harvest in virus-infected fruits. However, this protein has only been shown to be an important allergen for particular allergic subjects, prevalently living in the Mediterranean area [48]. Our subjects recruited from Berlin and surroundings might not be sensitised, and Lyc e 3 was never detected in one of our former studies with these subjects.

Besides the three already mentioned new putative tomato allergens identified in this study, seven additional candidates exist: a mannan endo-1,4-β-mannosidase, an enolase, a 1-aminocyclopropane-1-carboxylate oxidase homologue, a fructose-1,6-bishosphate aldolase, a nucleoside diphosphate kinase and consistently different heat shock proteins. The findings that heat shock proteins or superoxide dismutases could act as tomato allergens might be of particular interest in allergy research due to their wide distribution in nearly all studied organisms [49], [50]. However, further investigations with recombinant candidate proteins will be required to confirm them as true tomato allergens.

Taking a closer look at the 2DE gels the differences in the protein patterns are mainly due to the PepMV coat protein, only appearing in the gels of PepMV infected tomato fruits (arrows in Figure 2). Hence the PepMV coat protein has been indicated many times in database research after MS identification, along with other putative allergens. However, immunoblots with the purified PepMV coat protein revealed no reaction with the serum pool of tomato allergic subjects (data not shown).

### Clinical Allergy Tests with Tomato Allergic Subjects Revealed No Differences in the Reaction to PepMV Infected Tomato Fruits Compared to Non-infected Controls

Contrary to our hypothesis two standardised clinical allergy tests (skin prick test and basophil test) on tomato allergic subjects revealed no differences in reaction to PepMV infected and non-infected fruits, reflecting the results of the molecular analyses. To exclude possible variation of tomato fruits during the skin prick tests, basophil activation and degranulation tests were carried out at three different time points (4, 8, and 12 WPI). No significant differences could be detected, even if dose response curves tend to differ depending on time. In addition to fluctuations deriving from commercially cultivated tomato fruits, freshly harvested for every single subject, high inter-individual variation in subjects’ allergic reaction could be another reason for these results.

### Conclusions

This study shows for the first time that PR-protein and allergen transcript levels vary after viral pathogen attack in different tomato plant organs (leaves and fruits) weeks after inoculation with PepMV. Moreover, results from different time points and organs are non-transferable, which should be generally considered regarding the defence response of a plant at the RNA accumulation level. Additionally, clinical allergy tests showed high inter-individual variation to PepMV infected and non-infected tomato fruits. These inter-individual differences, and the fact that plants grown under commercial greenhouse conditions might differ regardless of the PepMV infection, makes it difficult to formulate a final statement about the allergenicity of PepMV infected tomato fruits.

The identification of ten new putative tomato allergens in this study reveals the wide spectrum of tomato allergens, and after more than ten years since identifying the first one, it seems that we are just at the beginning of understanding the allergenicity of tomato fruits.

## Supporting Information

Figure S1 **Relative PepMV quantification of tomato fruits and leaves 3 and 10 weeks post inoculation (WPI).** qRT-PCR analyses were carried out with primer pairs for genes encoding part of the PepMV genome. The target gene was normalised with a reference gene (*18S rRNA*). Data are given in CNRQ values (qBase software). Significant differences are indicated by asterisks (one-way ANOVA, p = 0.05; n = 3).
(TIF)

Figure S2 **PepMV infected tomato fruits at 3 and 10 weeks post inoculation (WPI).** Fruits showed typical PepMV symptoms (marbling) at 3 WPI, in contrast to fruits at 10 WPI, when no symptoms could be observed.
(TIF)

Figure S3 **Relative RNA accumulation of known and putative allergen encoding genes in tomato fruits.** RNA was extracted from tomato fruits of PepMV infected and corresponding non-infected plants 3 and 10 weeks post inoculation (WPI). qRT-PCR analyses were carried out with primer pairs for genes encoding the following proteins: Defence-related proteins: *Lyc e 3*: lipid-transfer-protein; *Lyc e 4*: from pathogenesis-related protein family PR-10; *GLU*: 1,3-β-glucanase; *CHI*: chitinase; *NP24*: thaumatin-like protein, osmotin precursor; *HSC70*: heat shock protein cognate; *PER*: peroxidase. Other confirmed and putative allergens: *Lyc e 1*: profilin; *Lyc e 2*: β-fructofuranosidase; *SOD*: superoxide dismutase; *CYC*: cyclophilin; *PG*: polygalacturonase; *PME2.1*: pectinmethylesterase 2.1; *PME1.9*: pectinmethylesterase 1.9; *MAN*: mannosidase; *VIC*: vicilin; *EXP*: expansin. Data were analysed using qBase software. Target genes were normalised with the geometric mean of three reference genes (*18S rRNA, GAPDH*, and *UBI*). CNRQ values and corresponding standard deviation of three replicates are shown. The table shows significant differences (*, p = 0.05; n = 3) calculated with factorial ANOVA. Interactions (PepMV*time point) and main effects (PepMV, time point) are shown.
(TIF)

Figure S4 **Relative RNA accumulation of defence-related allergen encoding genes in tomato leaves.** RNA was extracted from tomato leaves of PepMV infected and corresponding non-infected plants at 10 weeks post inoculation(WPI). qRT-PCR analyses were carried out with primer pairs for genes encoding the following proteins: *Lyc e 1*: profilin; *Lyc e 2*: β-fructofuranosidase; *Lyc e 3*: lipid-transfer-protein; *Lyc e 4*: from pathogenesis-related protein family PR-10; *GLU*: 1,3-β-glucanase; *CHI*: chitinase; *NP24*: thaumatin like protein, osmotin precursor; *HSC70*: heat shock protein cognate; *PER*: peroxidase; *SOD*: superoxide dismutase. Data were analysed using qBase software and calculated with CNRQ values. Target genes were normalised with the geometric mean of two reference genes (*18S rRNA* and *GAPDH*). Data are given in m-values (log_2_ (CNRQ +PepMV/CNRQ –PepMV)). Significant differences between PepMV infected plants and non-infected controls are indicated by asterisks (one-way ANOVA, p = 0.05; n = 3).
(TIF)

Figure S5 **Skin prick tests of single tomato allergic subjects with PepMV infected and non-infected control fruits.** Tests were carried out on nine subjects using tomato fruit mush from PepMV infected and non-infected control plants during the 4^th^–13^th^ week post inoculation (WPI). Histamine dichloride (10 mg/mL) was used as a positive control.
(TIF)

Figure S6 **Basophil activation and degranulation tests of single tomato allergic subjects with PepMV infected and non-infected fruits.** Basophil activation and degranulation is shown in %CD203c^+^ and %CD63^+^ cells normalised to a positive control. Tests from five tomato allergic subjects with tomato fruit protein extract from 4, 8 and 12 weeks post inoculation with PepMV (WPI) are shown.
(TIF)

Figure S7 **Basophil activation and degranulation tests of tomato allergic subjects with 5 µg/mL tomato protein extract of PepMV infected and non-infected fruits.** Basophil activation (A) and degranulation (B) tests with tomato fruit protein extract from 4, 8 and 12 weeks post inoculation with PepMV (WPI). Basophil activation and degranulation is shown in %CD203c^+^ and %CD63^+^ cells normalised to a positive controls. A median (black line) of five tomato allergic subjects is shown. No significant differences were found (Mann-Whitney U test, p = 0.05; n = 5).
(TIF)

Table S1 **List of primers for reference genes, PepMV detection/quantification and known and putative allergen encoding genes used in qRT-PCR.** The primers for target genes were designed using the DNAStar Primer Select software (GATC Biotech, Konstanz, Germany) based on tomato mRNA sequences deposited in NCBI database.*^1^: Mascia et al. [1], additionally tested with geNorm; *^2^: www.allergome.org; *^3^: detected on immunoblots.
(DOCX)

Table S2 **Tomato allergic subjects’ characteristics.** Abbreviations: m: male; f: female; n.d.: not done; b.d.: below detection limit; AD: atopic dermatitis; Bli: blister of the oral mucosa; D: dyspnoea; Er: facial erythema; mEr: mucosal erythema; GIT: symptoms of the gastro-intestinal tract including diarrhoea^1^, nausea^2^; OAS: oral allergic symptoms including numbness in the mouth^1^, burning tongue^2^, pruritus^3^, swelling lips^4^; eAD: exacerbation of atopic dermatitis.
(DOCX)
