# Supplementary figures and images for: Pepino mosaic virus Infection of Tomato Affects Allergen Expression, but Not the Allergenic Potential of Fruits
Source: PLoS One. 2013 Jun 7;8(6):e65116. doi: 10.1371/journal.pone.0065116 (PMC3676362; doi:10.1371/journal.pone.0065116)

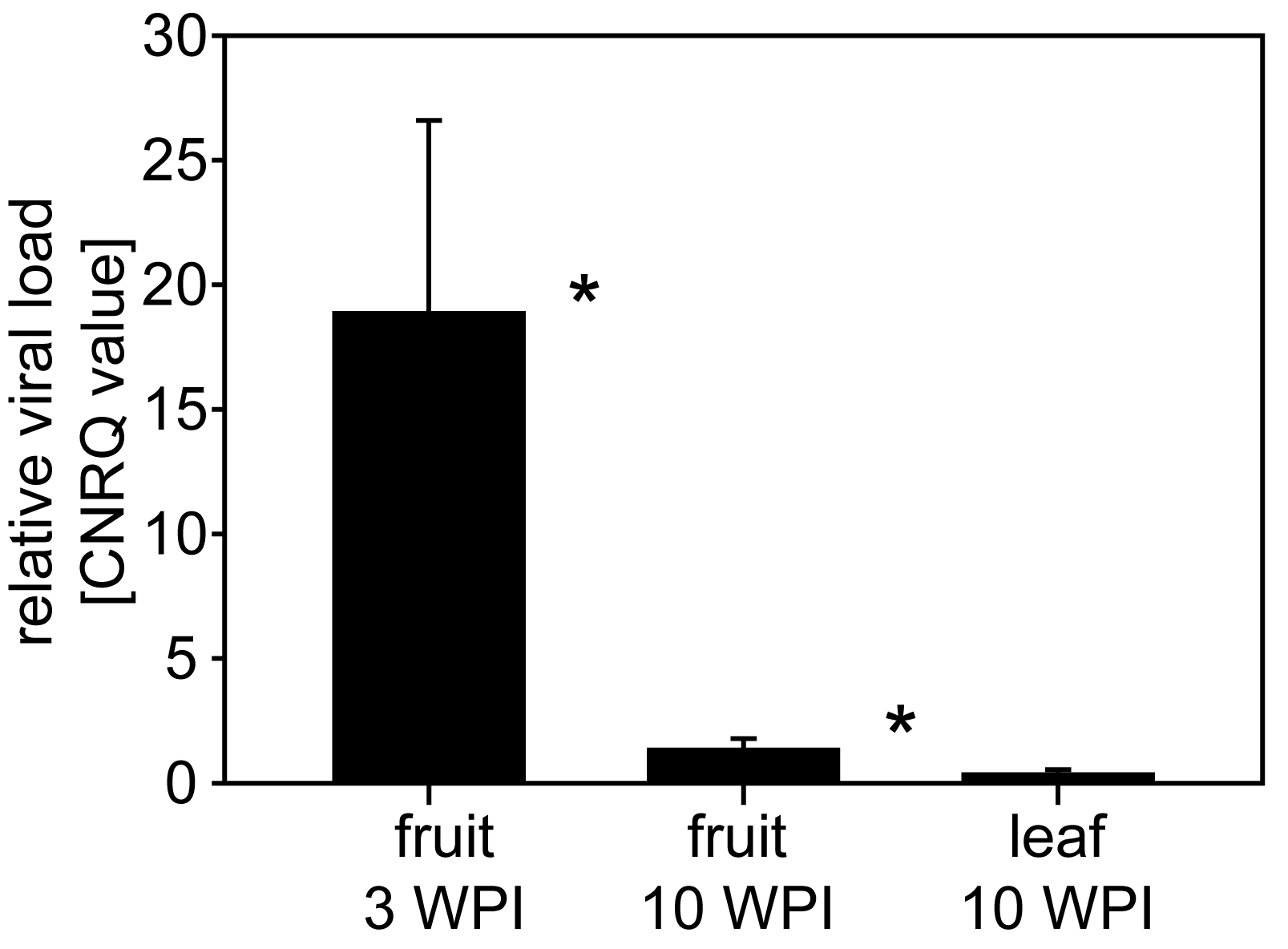

Supplement: Figure S1 — Relative PepMV quantification of tomato fruits and leaves 3 and 10 weeks post inoculation (WPI). qRT-PCR analyses were carried out with primer pairs for genes encoding part of the PepMV genome. The target gene was normalised with a reference gene (18S rRNA). Data are given in CNRQ values (qBase software). Significant differences are indicated by asterisks (one-way ANOVA, p = 0.05; n = 3). (TIF) [file pone.0065116.s001.tif]

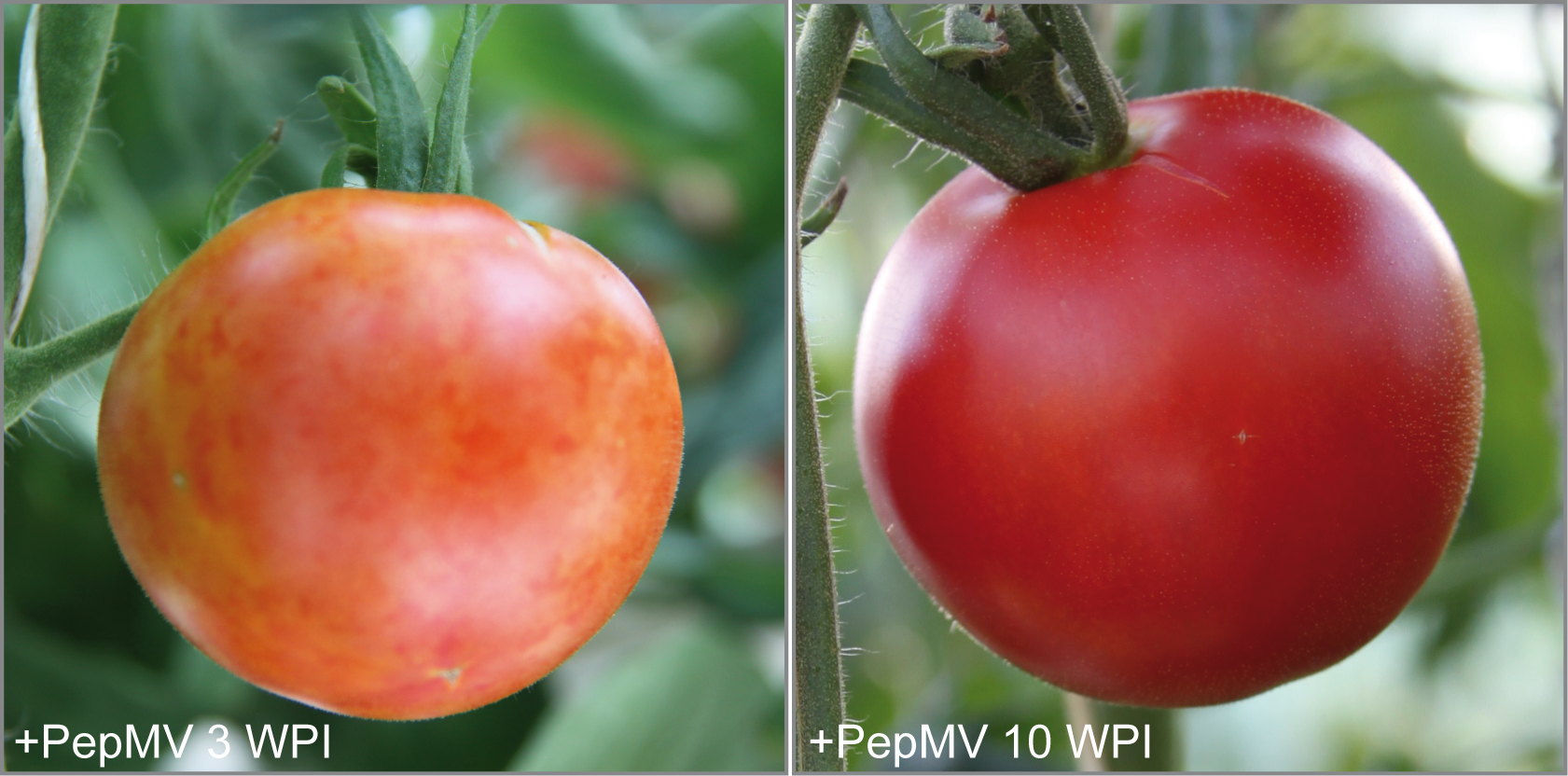

Supplement: Figure S2 — PepMV infected tomato fruits at 3 and 10 weeks post inoculation (WPI). Fruits showed typical PepMV symptoms (marbling) at 3 WPI, in contrast to fruits at 10 WPI, when no symptoms could be observed. (TIF) [file pone.0065116.s002.tif]

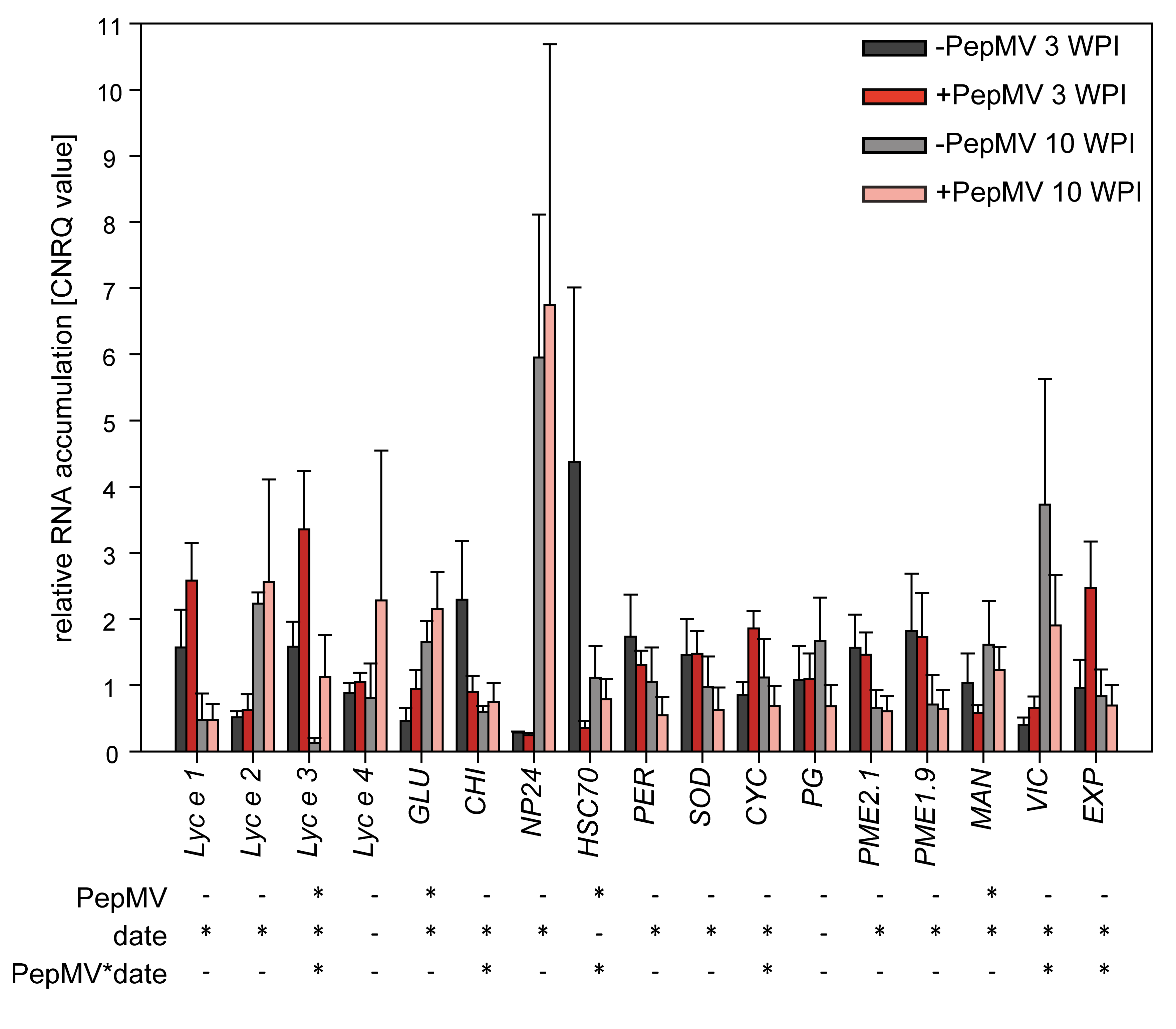

Supplement: Figure S3 — Relative RNA accumulation of known and putative allergen encoding genes in tomato fruits. RNA was extracted from tomato fruits of PepMV infected and corresponding non-infected plants 3 and 10 weeks post inoculation (WPI). qRT-PCR analyses were carried out with primer pairs for genes encoding the following proteins: Defence-related proteins: Lyc e 3: lipid-transfer-protein; Lyc e 4: from pathogenesis-related protein family PR-10; GLU: 1,3-β-glucanase; CHI: chitinase; NP24: thaumatin-like protein, osmotin precursor; HSC70: heat shock protein cognate; PER: peroxidase. Other confirmed and putative allergens: Lyc e 1: profilin; Lyc e 2: β-fructofuranosidase; SOD: superoxide dismutase; CYC: cyclophilin; PG: polygalacturonase; PME2.1: pectinmethylesterase 2.1; PME1.9: pectinmethylesterase 1.9; MAN: mannosidase; VIC: vicilin; EXP: expansin. Data were analysed using qBase software. Target genes were normalised with the geometric mean of three reference genes (18S rRNA, GAPDH, and UBI). CNRQ values and corresponding standard deviation of three replicates are shown. The table shows significant differences (*, p = 0.05; n = 3) calculated with factorial ANOVA. Interactions (PepMV*time point) and main effects (PepMV, time point) are shown. (TIF) [file pone.0065116.s003.tif]

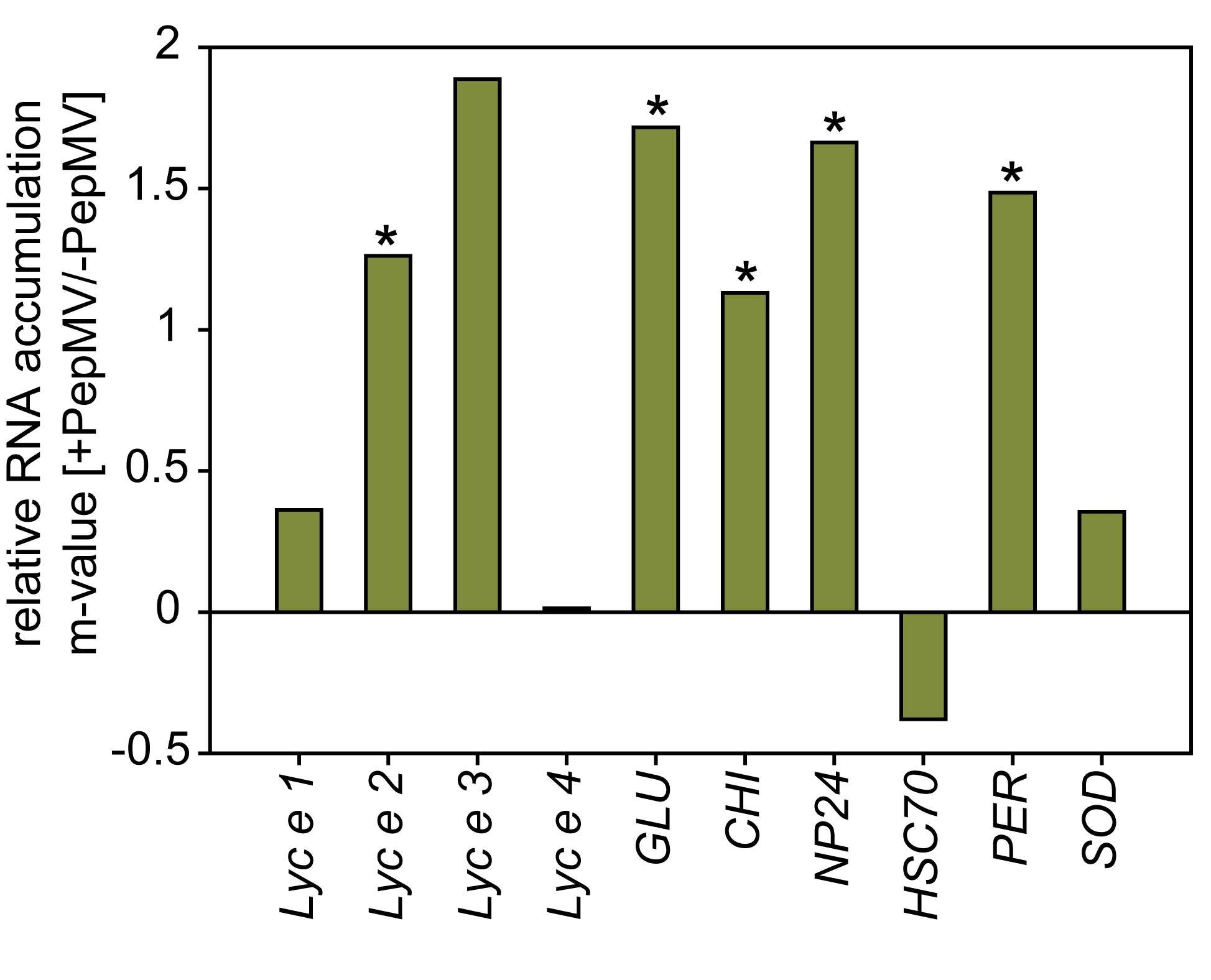

Supplement: Figure S4 — Relative RNA accumulation of defence-related allergen encoding genes in tomato leaves. RNA was extracted from tomato leaves of PepMV infected and corresponding non-infected plants at 10 weeks post inoculation(WPI). qRT-PCR analyses were carried out with primer pairs for genes encoding the following proteins: Lyc e 1: profilin; Lyc e 2: β-fructofuranosidase; Lyc e 3: lipid-transfer-protein; Lyc e 4: from pathogenesis-related protein family PR-10; GLU: 1,3-β-glucanase; CHI: chitinase; NP24: thaumatin like protein, osmotin precursor; HSC70: heat shock protein cognate; PER: peroxidase; SOD: superoxide dismutase. Data were analysed using qBase software and calculated with CNRQ values. Target genes were normalised with the geometric mean of two reference genes (18S rRNA and GAPDH). Data are given in m-values (log2 (CNRQ +PepMV/CNRQ –PepMV)). Significant differences between PepMV infected plants and non-infected controls are indicated by asterisks (one-way ANOVA, p = 0.05; n = 3). (TIF) [file pone.0065116.s004.tif]

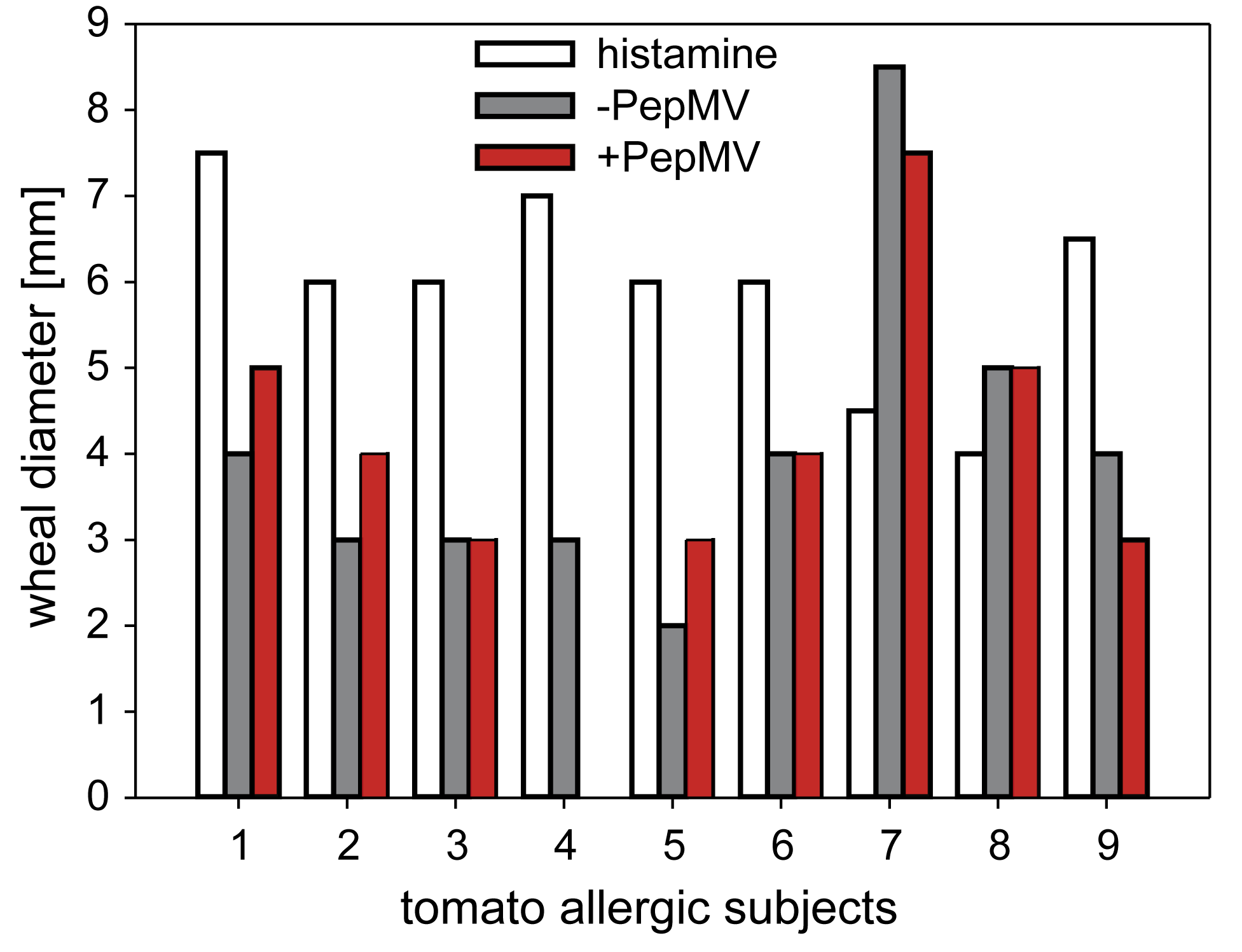

Supplement: Figure S5 — Skin prick tests of single tomato allergic subjects with PepMV infected and non-infected control fruits. Tests were carried out on nine subjects using tomato fruit mush from PepMV infected and non-infected control plants during the 4th–13th week post inoculation (WPI). Histamine dichloride (10 mg/mL) was used as a positive control. (TIF) [file pone.0065116.s005.tif]

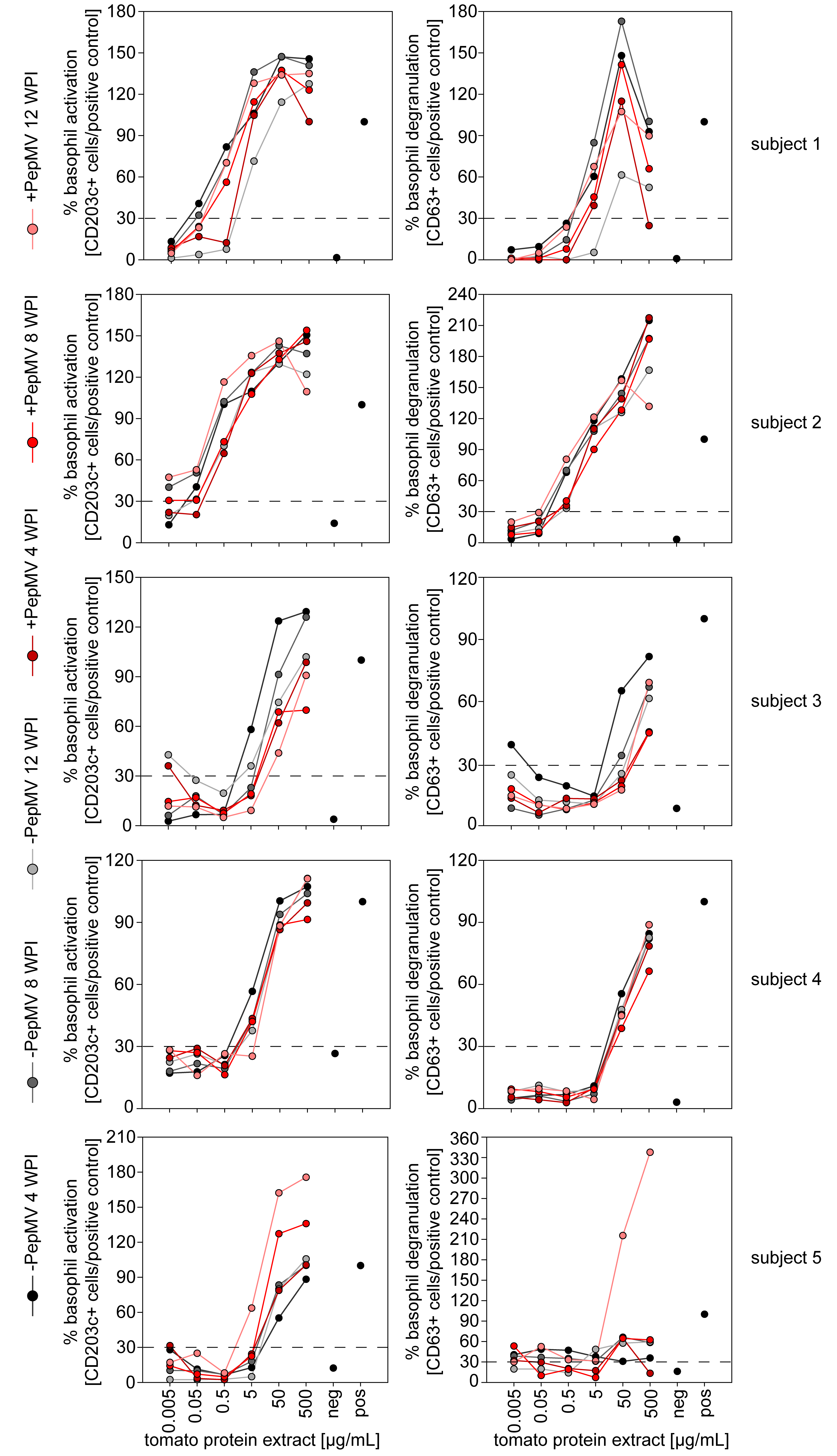

Supplement: Figure S6 — Basophil activation and degranulation tests of single tomato allergic subjects with PepMV infected and non-infected fruits. Basophil activation and degranulation is shown in %CD203c+ and %CD63+ cells normalised to a positive control. Tests from five tomato allergic subjects with tomato fruit protein extract from 4, 8 and 12 weeks post inoculation with PepMV (WPI) are shown. (TIF) [file pone.0065116.s006.tif]

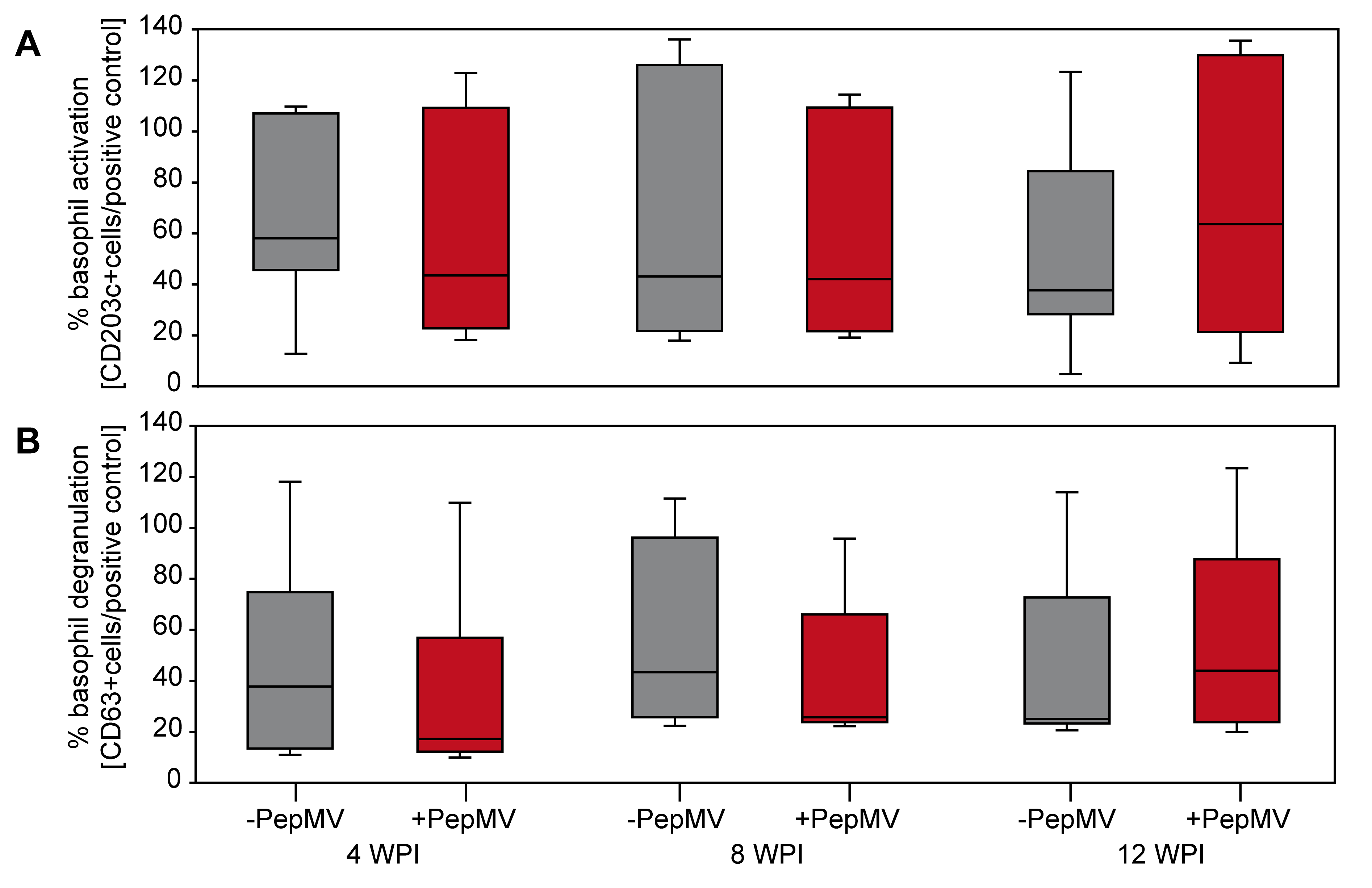

Supplement: Figure S7 — Basophil activation and degranulation tests of tomato allergic subjects with 5 µg/mL tomato protein extract of PepMV infected and non-infected fruits. Basophil activation (A) and degranulation (B) tests with tomato fruit protein extract from 4, 8 and 12 weeks post inoculation with PepMV (WPI). Basophil activation and degranulation is shown in %CD203c+ and %CD63+ cells normalised to a positive controls. A median (black line) of five tomato allergic subjects is shown. No significant differences were found (Mann-Whitney U test, p = 0.05; n = 5). (TIF) [file pone.0065116.s007.tif]
