# Supplementary material for: Pepino mosaic virus Infection of Tomato Affects Allergen Expression, but Not the Allergenic Potential of Fruits
Source: PLoS One. 2013 Jun 7;8(6):e65116. doi: 10.1371/journal.pone.0065116 (PMC3676362; doi:10.1371/journal.pone.0065116)
Supplement: Table S1 — List of primers for reference genes, PepMV detection/quantification and known and putative allergen encoding genes used in qRT-PCR. The primers for target genes were designed using the DNAStar Primer Select software (GATC Biotech, Konstanz, Germany) based on tomato mRNA sequences deposited in NCBI database.*1: Mascia et al. [1], additionally tested with geNorm; *2: www.allergome.org; *3: detected on immunoblots. (DOCX) [file pone.0065116.s008.docx]

**Table S1: List of primers for reference genes, PepMV detection/quantification and known and putative allergen encoding genes used in qRT-PCR.**

| name | NCBI accession number | primer sequence (5’-3’; for) | primer sequence (5’-3’; rev) |
| --- | --- | --- | --- |
| reference genes and PepMV detection | | | |
| *18S rRNA* (17S rRNA)*^1^ | X51576 | GTGCATGGCCGTTCTTAGTTGGTG | AAGAAGCTGGCCGCGAAGGGATAC |
| *GAPDH* (Glyceraldehyd 3-phosphate dehydrogenase)*^1^ | U93208 | ACCACAAATTGCCTTGCTCCCTTG | ATCAACGGTCTTCTGAGTGGCTGT |
| *UBI* (Ubiquitin)*^1^ | X58253 | TCGTAAGGAGTGCCCTAATGCTGA | CAATCGCCTCCAGCCTTGTTGTAA |
| PepMV detection | DQ000985 | GGAGCATTCATACCAAATGGG | CCTAGGTGAACCTATAACTAAG |
| Confirmed and potential allergens | | | |
| *Lyc e 1* (profilin)*^2^ | AY061819 | TGGGCTCAATCTGCTAAT | AGTCATCGGCTCGTCATA |
| *Lyc e 2* (beta-fructofuranosidase)*^2^ | AF465612 | CGTACCCCGCCAACTTATCTG | CAATGCCGGGTGGAGGAA |
| *Lyc e 3* (non-specific lipid transfer protein)*^2^ | AM051295 | TGGCTCCTTGTCTCCCTTATCT | TTGCCCAAATTGAGTCCTGTA |
| *Lyc e 4* (TSI 1 protein)*^2^ | Y15846 | AACCACAATTTCCCCAACAAG | CCACCATCTCCCTCAATAGTCTC |
| *GLU* (beta-1,3-glucanase)*^2^ | M80608 | TCAAACATCATGGCTACCTCACAA | TCCCCATCATTCCATAACAAACAC |
| *CHI* (chitinase)*^2^ | Z15140 | ATGGGGTTACTGTTTCCTTAGAGA | TGGCCCATAGTTGTAGTTGTGT |
| *NP24* (PRP osmotin precursor)*^2^ | AF093743 | TATATGGGGTCGTACTGGTTGC | GCTAGGGTGTTTGGGGGTTTG |
| *HSC70* (heat shock protein cogante 70)*^3^ | L41253 | TTCTGTGATACTTTTGCTTTACTA | TGCTGCCATACACCTACAC |
| *PER* (anionic peroxidase)*^2^ | X15853 | ATTAATTAAACCTGGCCGTATGAT | ATTAATTAAACCTGGCCGTATGAT |
| *SOD* (superoxide dismutase)*^3^ | M37150 | TCGCCGTCCTTAACAGCAGTG | AAGCCATGAAGTCCAGGTTTTAGG |
| *CYC* (cyclophilin)*^3^ | M55019 | TGGATGTGATTAAGAAGGCAGAGG | GACCCGACCAAAGCAGTAGAGATA |
| *PG* (polygalacturonase 2a)*^2^ | A15981 | CAGGTGATGATTGTATTTCAATTGTTT | CCATGACCTGGACCACAAGTAA |
| *PME1.9* (pectin methylesterase)*^2^ | U50986 | CCCGCTAAAGCTATCCCGTTCA | GTTATGCTTGCTCTGCCCTGCTC |
| *PME2.1* (pectin methylesterase)*^2^ | U50985 | GGGCTGAGTGGCACGGAGATT | CCAGGCCACTTGACACGCTTACT |
| *MAN* (beta-mannosidase)*^3^ | AF403444 | GTTGGGCCGTGAAGTCGTGAA | CGTACCCCAAGGCAGCAACC |
| *VIC* (putative vicilin)*^2^ | AJ270964 | GCCTCGCGCTCAACTCAG | TTCGGGACGTGCTTCAAA |
| *EXP* (expansin)*^3^ | U82123 | TAACCCTCCTCGCCCTCACTTT | TATCCCCTGCACCTGCTACATTC |

The primers for target genes were designed using the DNAStar Primer Select software (GATC Biotech, Konstanz, Germany) based on tomato mRNA sequences deposited in NCBI database.*^1^: Mascia et al. [1], additionally tested with geNorm; *^2^: www.allergome.org; *^3^: detected on immunoblots.

Reference:

1. Mascia T, Santovito E, Gallitelli D, Cillo F (2010) Evaluation of reference genes for quantitative reverse-transcription polymerase chain reaction normalization in infected tomato plants. Molecular Plant Pathology 11: 805-816.
