# Supplementary material for: Pepino mosaic virus Infection of Tomato Affects Allergen Expression, but Not the Allergenic Potential of Fruits
Source: PLoS One. 2013 Jun 7;8(6):e65116. doi: 10.1371/journal.pone.0065116 (PMC3676362; doi:10.1371/journal.pone.0065116)
Supplement: Table S2 — Tomato allergic subjects’ characteristics. Abbreviations: m: male; f: female; n.d.: not done; b.d.: below detection limit; AD: atopic dermatitis; Bli: blister of the oral mucosa; D: dyspnoea; Er: facial erythema; mEr: mucosal erythema; GIT: symptoms of the gastro-intestinal tract including diarrhoea1, nausea2; OAS: oral allergic symptoms including numbness in the mouth1, burning tongue2, pruritus3, swelling lips4; eAD: exacerbation of atopic dermatitis. (DOCX) [file pone.0065116.s009.docx]

**Supplemental table S2: Tomato allergic subjects’ characteristics.**

| **subject**  **no.** | **age**  **[years]** | **sex** | **sIgE tomato**  **[in kU/l]** | **tomato allergy**  **symptoms** |
| --- | --- | --- | --- | --- |
| 1 | 33 | m | n.d. | OAS^3^, GIT^2^, D  (only anamnesis) |
| 2 | 28 | m | 1.04 | mEr, Bli, GIT^1^ |
| 3 | 38 | f | 0.37 | OAS^1^, mEr, GIT^1^ |
| 4 | 59 | f | b.d. | OAS^2^, mEr |
| 5 | 43 | f | 1.17 | OAS^3^, GIT^2^, Er |
| 6 | 57 | f | 2.55 | OAS^2^, Er |
| 7 | 31 | m | 9.57 | OAS^2,3^, Er |
| 8 | 46 | f | 0.47 | OAS^3^, mEr |
| 9 | 32 | m | 42.9 | eAD, OAS^4^  (only anamnesis) |

Abbreviations: m – male, f – female, n.d. – not done, b.d. – below detection limit, AD – atopic dermatitis, Bli – blister of the oral mucosa, D – dyspnoea, Er – facial erythema, mEr – mucosal erythema, GIT incl. diarrhoea^1^, nausea^2^, OAS – oral allergic symptoms incl. numbness in the mouth^1^, burning tongue^2^, pruritus^3^, swelling lips^4^; eAD – exacerbation of atopic dermatitis
